# rosettR: protocol and software for seedling area and growth analysis

**DOI:** 10.1186/s13007-017-0163-9

**Published:** 2017-03-15

**Authors:** Filipa Tomé, Karel Jansseune, Bernadette Saey, Jack Grundy, Korneel Vandenbroucke, Matthew A. Hannah, Henning Redestig

**Affiliations:** 1 Bayer CropScience NV, Technologiepark 38, 9052 Ghent, Belgium; 2 Max Planck Institute for Plant Breeding Research, 50829 Cologne, Germany; 3 Cluster of Excellence on Plant Sciences “From Complex Traits towards Synthetic Modules”, 40225 Düsseldorf, Germany; 4 DTU Biosustain, Kemitorvet, Building 220, 2800 Kgs. Lyngby, Denmark; 5 School of Life Sciences, University of Warwick, Coventry, CV4 7AL UK

**Keywords:** Phenotyping, R, Growth, Leaf area, Image analysis

## Abstract

**Background:**

Growth is an important parameter to consider when studying the impact of treatments or mutations on plant physiology. Leaf area and growth rates can be estimated efficiently from images of plants, but the experiment setup, image analysis, and statistical evaluation can be laborious, often requiring substantial manual effort and programming skills.

**Results:**

Here we present *rosettR*, a non-destructive and high-throughput phenotyping protocol for the measurement of total rosette area of seedlings grown in plates in sterile conditions. We demonstrate that our protocol can be used to accurately detect growth differences among different genotypes and in response to light regimes and osmotic stress. rosettR is implemented as a package for the statistical computing software R and provides easy to use functions to design an experiment, analyze the images, and generate reports on quality control as well as a final comparison across genotypes and applied treatments. Experiment procedures are included as part of the package documentation.

**Conclusions:**

Using rosettR it is straight-forward to perform accurate, reproducible measurements of rosette area and relative growth rate with high-throughput using inexpensive equipment. Suitable applications include screening mutant populations for growth phenotypes visible at early growth stages and profiling different genotypes in a wide variety of treatments.

## Background

The study of how different genes influence observable traits and particularly growth patterns is a key interest in plant science. Knowledge of the importance of a gene for the plant’s ability to cope with stress can help us understand the molecular background of adaptation mechanisms [1, 2]. From an applied perspective, genes that are linked to enhanced growth rate may be used to develop genetically modified (GM) traits or to design genetic markers for breeding programs [3, 4].

Our ability to generate data on the expression and regulation of genes has increased dramatically following the rapid development of high-throughput sequencing technologies [5]. However, in order to draw connections between genes and traits, it is crucial to couple molecular information with corresponding physiological data. As generating phenotyping data is a much more diverse topic than sequencing, available genomic information is still far from being fully exploited for discovering gene-trait associations [6, 7]; acquiring reliable and accurate phenotypic data in a high throughput manner remains a challenge. However, the quantification of leaf area and growth analysis is extremely useful to experimentally identify potential candidate targets and has already been used to identify genes involved in for example drought stress [8, 9] or salt tolerance [10].

Numerous phenotyping platforms have been developed for several traits with varying levels of automation and throughput [11–14], and there is continued pressure to develop further solutions [15, 16]. Phenotyping projects of sufficient size and budget can be carried out in collaboration with scientific networks such as the European Plant Phenotyping Network [17] and the Australian Plant Phenomics Facility [18]. However, for small projects it is often preferable to use solutions that can be carried out on-site.

In our research on the genetic factors underlying plant growth we wanted to screen the growth phenotype of a large number of different Arabidopsis genotypes under both favorable and stress inducing conditions. We decided to first perform a pre-screen in a sterile in vitro environment in order to reach a more manageable number of genotypes to be tested on soil in a greenhouse, as that necessarily requires more effort and growth space. To support this screen, we required a phenotyping solution based on image analysis that could estimate relative growth rates in a non-destructive manner. As we expected differences between genotypes to be subtle, we required large numbers of replicates to achieve sufficient statistical power, yet still with minimal space requirements to allow for concurrent experiments. Furthermore, the protocol needed to be highly automated and reproducible to enable multiple team members to collaborate easily.

Available tools including LAMINA [19], LeafAnalyser [20], LeafJ [21], Easy Leaf Area [22], and Black Spot [23] can successfully calculate a wide variety of shape parameters by analyzing images of individual leafs or whole plants. Shoot architecture and morphology can be investigated with tools such as Phytotyping4D using advanced light-field cameras that is particularly suitable for developed plants [24].

However, as our time-series experiments resulted in thousands of data points (e.g. 20 genotypes, 2 treatments, 80 biological replicates, 4 time-points = 12,800 seedling images) we needed a packaged solution that could track rosette areas and identities of several in situ seedlings in the same image cross-referenced with the experiment design. We furthermore needed software to facilitate experiment design and performing statistical analysis. As we could not find an existing solution that fit our needs, we developed *rosettR*. We chose to implement rosettR as a package for R [25] as that enabled fast development, easy automation, and access to the huge library of other packages for graphics and statistical analysis.

For the image analysis steps, we opted to use EBImage [26] which is a general purpose image analysis package for R. Using EBImage, we developed algorithms to estimate the areas of 32 individual seedlings growing together on a tissue culture plate, addressing aspects such as finding the plate, adjusting for rotation, and allowing for seedlings to partially grow outside their designated area. We then bundled this analysis with web-based template reports using the excellent knitr package [27] to present experiment design, quality control of the image analysis, and a final comparison across genotypes and applied treatments.

Our expectation is that linking phenotyping and molecular data is facilitated by phenotyping solutions that allow for fast measurement of traits of interest in a way that can be preliminary but still accurate and informative. As we found rosettR to be very useful, we made it available as free/open source software in the hope that other plant researchers may benefit from it as well. In this paper we present the experimental protocol and image analysis implemented in rosettR and demonstrate the applicability by describing two different use cases. rosettR is available on github [28] including detailed documentation and a step-by-step user guide.

## Methods

### rosettR: a tool for screening seedling areas and growth rate

**Fig. 1:**
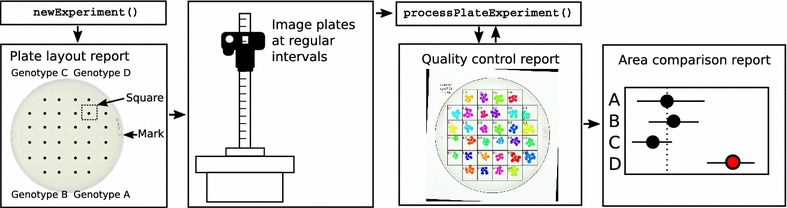
Overview of rosettR phenotyping protocol. The sowing is facilitated by the auto-generated plate layout report. When all images are available, the images are analyzed and a quality control report is generated. Results are provided as spreadsheet with estimated areas and a report with statistical evaluation of genotype and treatment effects

rosettR is a phenotyping protocol for tracking the growth of Arabidopsis seedlings over time (Fig. 1). The prerequisites for using rosettR are a fixed digital camera, a growth chamber, tissue culture plates, medium, a computer, the freely available software R [25], and the seeds of the genotypes to test.

**Fig. 2:**
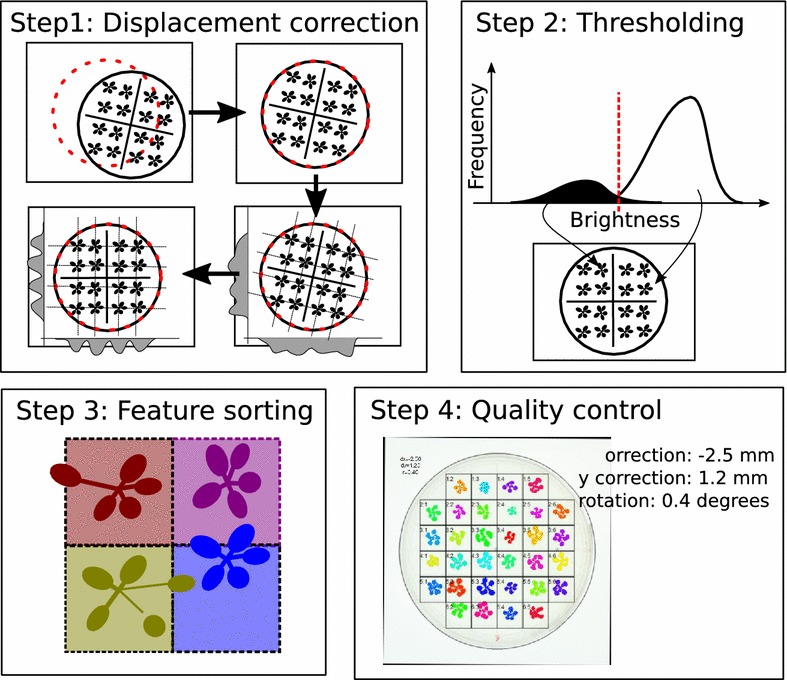
Overview of image analysis steps in rosettR. *Step 1:* eccentricity of the plate is corrected for using an optimization algorithm that ensures that as many dark pixels as possible are within a circle matching the size of the plate. Rotation is corrected for by searching for clear peaks in the marginal distributions when step-wise rotating the image. *Step 2:* establishing a suitable threshold for background is done by a 2-component mixture model classifying pixels to the bright (backlit) background or all other darker pixels as plant material. *Step 3:* detected image features are sorted to the square in the grid they occupy the most. *Step 4:* a quality control image is generated that indicates the correction measures and colors the image features to indicate if they were detected correctly

Images of the plates with the growing seedlings are taken at regular intervals, and the rosette areas are estimated from the images, requiring minimal user interaction. Template reports are generated to support sowing, quality control, and area and growth rate comparisons for each experiment. The protocol is compatible with a wide range of stress treatments applied at any desired time point during the experiment. Treatments such as different light regimes or temperatures can be applied by simply shifting the plates to the desired condition. Other treatments can be applied from germination by supplementing the medium with sugars, sorbitol, salt, among others. Another option is to place a membrane on the solid media and sow directly on top of the membrane. The seeds/seedlings and the membrane can be transferred to new plates with the desired supplement at a later stage of the experiment.

### Starting a new experiment

A new experiment can be started by loading the package in R and providing information regarding the genotypes, treatments, time-points at which pictures will be taken, the number of repetition blocks, and the genotype to use as reference. The package provides high-level functions to create a directory tree where images are to be placed and reports that define the randomized block design to facilitate sowing and the placement of the plates in the growth chamber. Images are then taken at the pre-defined time-points and saved in the corresponding directory. Once all images have been taken, they can be analyzed to compute areas and relative growth rates.

The tissue culture dishes we used are 150 × 25 mm and have a grid that divides the plate in 32 squares. A single seed is placed in each square, so each plate can have a total of 32 seedlings from different genotypes. Plants from different genotypes are sown on the same plate in alternating combinations to account for differences between plates.

Half-strength Murashige and Skoog media with 1% glucose is poured in the plates in sterile conditions, and the sterile seeds placed on the solid medium with a Vacuumseed or sterile toothpick/pipette in the corresponding square. The plates are then sealed with Urgopore tape, wrapped in groups of 10 with transparent foil, and placed at 4 °C in darkness for three nights for seed stratification. Plates are placed on the shelf in a growth chamber, and the plants allowed to grow at a temperature of 20–22 °C and 150 μmol m^−2^ s^−1^ light intensity.

**Fig. 3:**
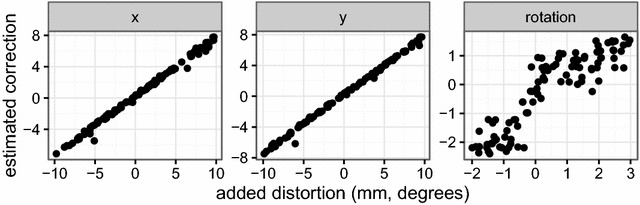
Simulation test for correction of plate location and rotation. We applied known dislocation in x/y direction and rotation to 100 images and then tried to re-cover the original image using our correction steps. The exact center (x- and y-coordinate) of the location can be correctly identified as shown by the good correspondence between simulated dislocation and estimated correction. Plate rotation is hard to determine exactly but in our experience sufficiently accurate

**Fig. 4:**
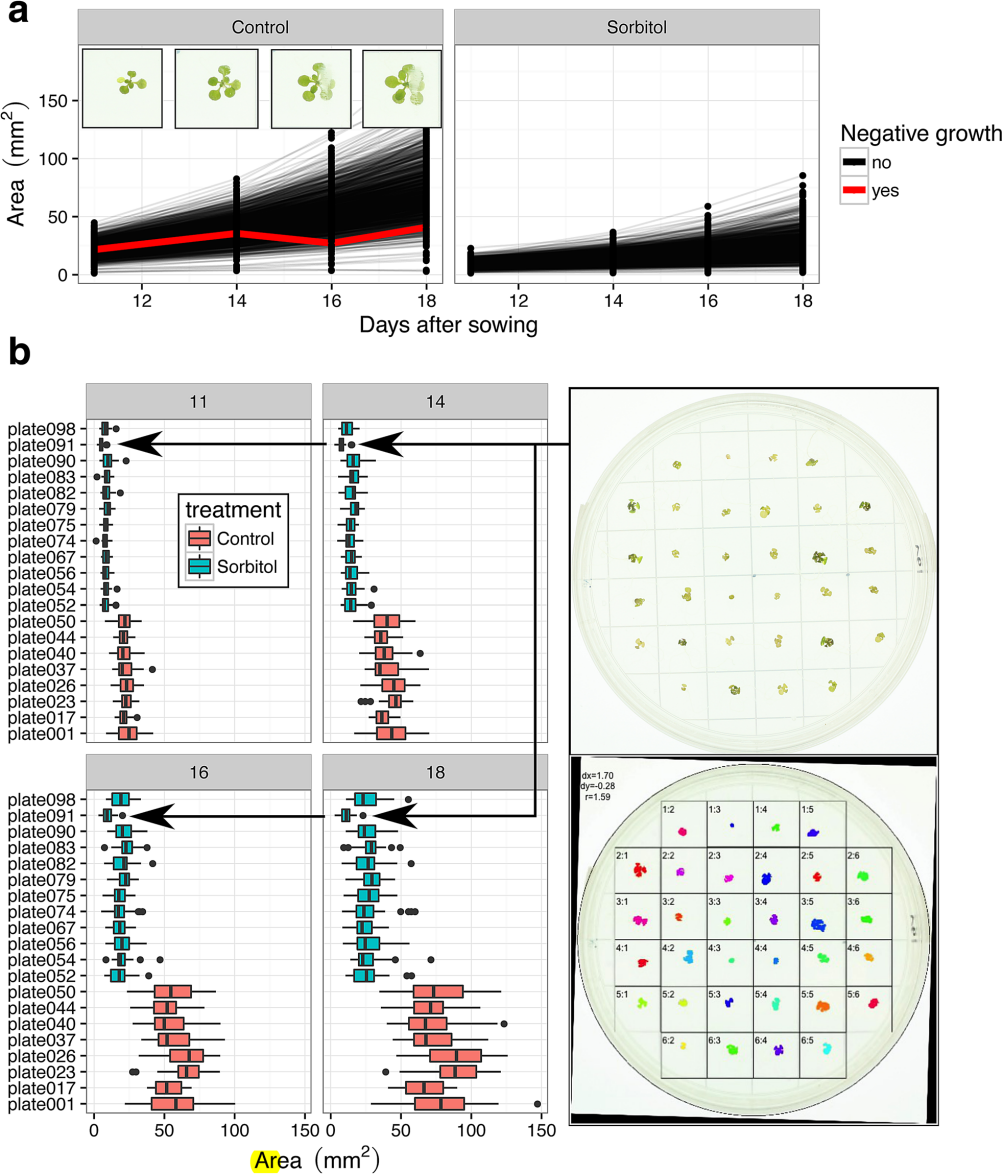
Plots used for quality control. **a** A *line plot* of the growth curves for all individual plants faceted on the applied treatment. Plants with negative derivatives in the growth curves are usually not detected correctly and may be excluded. Here, condensation had formed in one plate obscuring a seedling resulting in inaccurate area estimation. **b** *Boxplots* with plant areas per day. Plates with outliers or very large or small areas may have technical problems. In this case, seedlings in plate091 are much smaller than all other plants. The quality control image shows that the seedlings were accurately detected suggesting a technical problem with the growing conditions of for this particular plate. Thanks to the high replication, such extreme plates can safely be left out from the analysis

Images should have a uniform bright background to avoid any shadows and allow for accurate detection of the seedlings. We recommend using a white backlit imaging table with homogeneous background, avoiding any formation of shades, and the camera mounted firmly right above the plates for the whole duration of the experiment. Preferably, the imaging is done inside the growth chamber to avoid temperature differences that may cause condensation on the lid of the plate or affect experiment treatments.

### Image analysis

All images are expected to be taken with the same zoom factor which is manually defined by indicating two points on an image at a set distance in millimetres using the calibrateScale function. After that, the remaining estimation and recording of rosette areas for each seedling is achieved by a fully automated workflow depicted schematically in Fig. 2.

*Step 1: Displacement correction* Seedlings are expected to be sown in a pre-defined grid of configurable dimensions. The first step of the image analysis is to make sure that the grid is centered and at a right angle to the image edges. The exact x- and y-coordinate of the plate centers are determined using a Nelder and Mead [29] optimization algorithm that maximizes the fraction of dark pixels (plate) to light pixels (background) within the circle. Plate rotation is compensated by applying step-wise rotation of the image at a given interval (e.g. −5°:5°), and by interpolation choosing the angle that minimizes the common standard deviation of a multi-component normal distribution with means at the centres of each square in the grid. Conceptually, this can be thought of as moving along a forest with trees planted in rows, and then stopping when all trees align and one can see the other side of the forest. In order to test these correction steps, we performed a small simulation study applying known dislocation of the plate as well as small rotation to 100 images. The algorithm could accurately recover the displacement in horizontal (x) and vertical (y) direction (Fig. 3). Detecting the rotation was less precise but still of sufficient accuracy to correctly identify the grid in the corrected image.

**Fig. 5:**
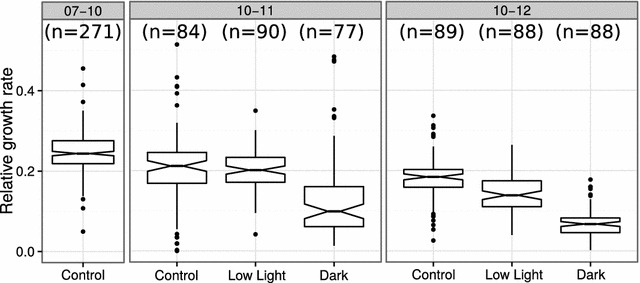
Growth response of wild-type Arabidopsis Columbia to different light regimes, in 16 h light/8 h dark. Between days 7 and 10 all plants were in control conditions (150 μmol m^−2^ s^−1^ light intensity). Low light (50 μmol m^−2^ s^−1^ light intensity) and dark were applied at day 10 for 24 (time interval 10–11) or 48 h (time interval 10–12) by shifting the plates to the respective conditions. Control plates remained in original conditions and were not shifted. The data used for the *boxplot* was obtained from the spreadsheet with estimated areas and relative growth rates for each plant, provided after compiling the template compare areas report

**Fig. 6:**
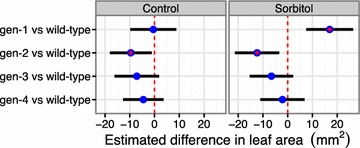
Example from the compare areas report. The x axis indicates the estimated difference between the leaf area of the wild-type and the tested genotypes. Gen-1, gen-2, gen-3, and gen-4 correspond to SALK_092889, SALK_057095, SALK_046986, and SALK_007071, respectively. The *red dot* indicates statistical significance (ANOVA *t* test *p* ≤ 0.05). Gen-1 is the best candidate for further studies as it behaves in a similar way to the wild-type in control conditions, but has a larger leaf area when treated with sorbitol for 18 days

*Step 2: Thresholding* The default behavior of rosettR is to convert images to greyscale by keeping only the blue channel as healthy plant material absorb blue light thereby appearing dark. For seedlings with strong discoloration, the weighting of the three channels (red, green, blue) can be adjusted by the user. A threshold for segmenting the plate in foreground (seedling) and background (plate) is determined by fitting a mixture model of two normal distributions truncated at 0 and 1 to the plate region of the image, yielding a tuned threshold per image. In our experience, this works well since the background is bright and homogeneous resulting in a distinct class of bright pixels, whereas all darker pixels can be assumed to be the seedling.

*Step 3: Sorting image features* Image features are identified using the bwlabel function in EBImage [26] and each feature is sorted to the square it occupies the most. The area of each plant is then finally estimated as the sum of all image features allocated to each corresponding square using the computeFeatures.shape function. This sorting procedure implies that leaves that are detected as detached from the rosette but still mostly in the right square, or still attached to the seedling but predominantly in the wrong square, will still be classified to the right seedling even if it reaches into the neighboring square.

**Fig. 7:**
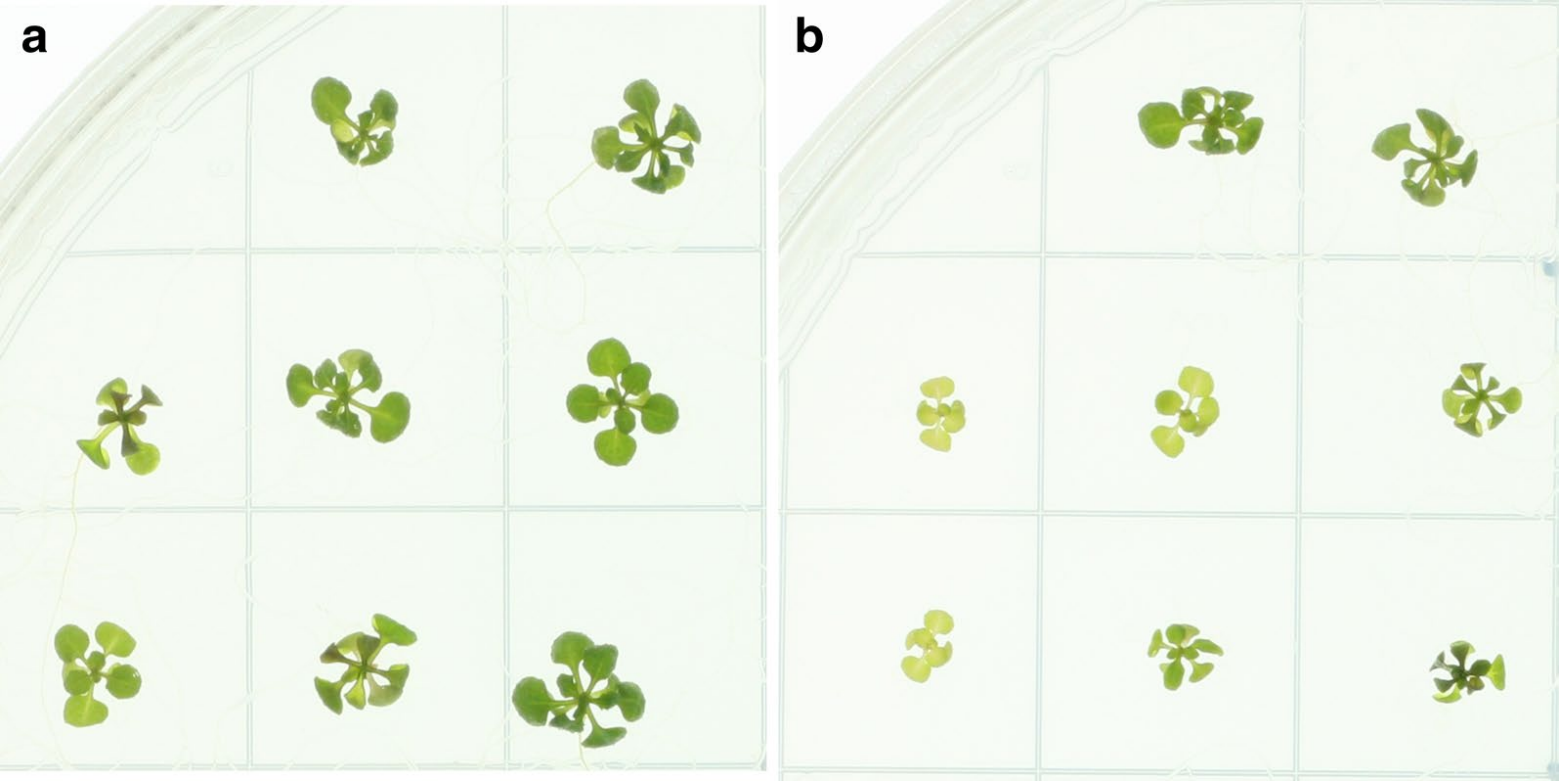
Eight mutant (**a**) and eight wild-type plants (**b**) in sorbitol conditions, 18 days after stratification. The rosette areas of the mutant lines are larger than the wild-types

*Step 4: Quality control* Finally, a quality control image is generated for each plate where the outline of the plate and each square is indicated [see example in Fig. 4 (Step 4)]. Features for the same plant are colored with the same color and squares where plants had been found to be merged between squares are outlined red (not shown in the figure). Plants residing in such *ambiguous squares* are marked in the final data sheet and can be ignored during comparisons of plant areas and relative growth rates.

Once image analysis has finished, a template report can be compiled that shows all quality control images in a convenient overview, as well as growth curves and boxplots highlighting plates with ambiguous squares or outliers (Fig. 4).

### Data analysis and visualization

The main output of the image analysis is a spreadsheet with estimated areas and relative growth rates for each plant. With this data, it is straight-forward to perform statistical analysis to compare genotype and treatment effects as needed given the exact context of the experiment. For comparison between examined genotypes and a reference of choice, and as an example in general, the *compare areas* template report can be generated non-interactively after successfully completed image analysis. For estimation of effect sizes and significance, we use a 2-way ANOVA (genotypes and treatments) and the multiple comparisons framework described in [30]. An example of an area comparison report can be seen at [31].

## Results

In order to illustrate the applicability of rosettR, we provide below two examples where our protocol was used to characterize the physiological response of different Arabidopsis genotypes to two stress conditions. The objective was to demonstrate that the stresses applied result in a decreased relative growth rate, which is estimated by measuring the leaf area of the seedlings over time.

### Low light and dark treatment

We wanted to characterize the response to light deprivation for several different Arabidopsis genotypes by subjecting them to different light regimes and evaluating their relative growth rate. The first step was to test several light intensities and durations of treatments to identify the most suitable to screen a large number of genotypes. In order to increase the number of treatments and replicates per experiment, we chose to reduce the number of genotypes and therefore only the reference genotype Columbia was used in the initial tests that we present here. For this purpose, we initialized a rosettR experiment with imaging on day 7, 10, 11 and 12. Seeds were sown on plates, stratified for three nights, and then allowed to germinate and grow for 10 days in control conditions. Between day 7 and 10, the estimated relative growth rate was similar to what has been described in the literature [32]. At day 10 the plates were shifted to dark or low-light regimes for 24 or 48 h, and then returned to control conditions. Control plants remained in the same light conditions for the whole duration of the experiment. The low-light treatment for 24 h did not have a measurable effect on leaf area, but 48 h of low light caused a reduction in relative growth rate (Fig. 5). The dark treatment for 24 and 48 h caused a reduction in the relative growth rate, and this effect was stronger when the dark treatment was applied for 48 h (Fig. 5). In summary, the treatment had an effect in the leaf area of the studied genotype, translated into reduced relative growth rates that could be detected by rosettR. As the 48 h low light and dark treatment had a stronger growth response phenotype, we used these treatments to phenotype a large number of mutants targeting candidate genes related to light deprivation responses.

### Osmotic stress

We have used rosettR to test candidate genes that have been hypothesized to be related to the response to osmotic stress. In these trials, Arabidopsis wild-type lines and T-DNA insertion mutants targeting candidate genes (SALK_092889, SALK_057095, SALK_046986, and SALK_007071) were sown on plates with solid media supplemented with 100 mM sorbitol to induce osmotic stress. After sowing and seed stratification, the plates were placed in the growth chamber and images were taken at days 11, 14, 16, and 18, and analyzed using rosettR. The aim of the experiment was to identify mutants with larger leaf areas than the wild-type in sorbitol conditions at the end of the experiment, and similar performance in control conditions. One out of the four genotypes tested, SALK_092889 (gen-1) fits these criteria (Fig. 6), also visible by inspecting an example plate (Fig. 7). Gen-2 (SALK_057095) showed reduced leaf area than the wild-type in both conditions, suggesting that this reduction is not a treatment effect but likely a genotype effect. Gen-3 and -4 (SALK_046986, SALK_007071) show similar leaf area when compared to the wild-type in both conditions, suggesting there is no treatment or genotype effect (Fig. 6). Gen-1 corresponds to the most interesting line for further study due to its better growth under sorbitol stress conditions, and interestingly this is in agreement with a previous publication reporting reduced sensitivity to drought stress for this T-DNA allele [33].

Here, rosettR thereby helped to provide experimental support to our hypothesis by identifying a mutant that reached a larger rosette area than the wild-type when treated with sorbitol. The gene affected in this mutant is a suitable candidate for further testing and investigation.

## Discussion

Stress, different treatments, and mutations in genes that affect overall plant physiology in a given environment frequently influence the growth of the plant making it a useful indicator trait [34]. Growth can be quantified by several different parameters such as fresh or dry weight, height, leaf area, or metabolite content [35, 36]. Leaf area is both easy and fast to measure in a non-destructive fashion allowing for time series experiments [37]. Here we presented rosettR, a phenotyping protocol suitable for a primary screen on the growth performance of a large number of genotypes and treatments with high replication, and with few resource requirements making it easy to carry out in any plant research laboratory.

In contrast to the many reported general image analysis tools for plant phenotyping, rosettR provides a packaged solution for all steps of a protocol for phenotyping, from experiment design to the final rosette area and relative growth rate comparisons. Other tools available can also estimate leaf areas from images of scanned leaves or rosettes, but to the best of our knowledge rosettR is the only tool that provides both image and data analysis and at the same time is affordable and easy to setup in any laboratory (for a schematic comparison between rosettR and other available tools, see Table 1). rosettR is particularly suitable for screening large populations of seedlings in response to treatments applied in the growth medium or application of different light or temperature regimes for growth related phenotypes visible at the seedling stage.

**Table 1:** Comparison of rosettR with other available tools in the literature

Tool	Input	Rosette detection	Data analysis	Easy in house setup	Multiple traits	References
Easy leaf area	Single	Automatic	No	Yes	No	[22]
Black spot	Single	NA	No	Yes	No	[23]
Rosette tracker	Single/multiple	Manual^a^	No	Yes	Yes	[41]
Phenotyping pipeline for Arabidopsis	Single	Automatic	Yes	No	No	[42]
rosettR	Multiple	Automatic	Yes	Yes	No	This study

Input is in all cases images of seedlings. Some software can handle multiple seedlings in the same image (single/multiple). All tools mentioned are free/open source software. With *data analysis* is meant if the software includes calculation of statistics to allow inference of genotype/treatment effects directly

^a^Rosette tracker is automated when there is only one seedling per image. With more, the user must keep track of identity of seedlings interactively

The inherent in vitro nature of this assay meant that we could miniaturize our screens in order to allow for large numbers of biological replicates. For example, by sowing 32 seedlings on a single tissue culture plate we can easily track thousands of seedlings in a single experiment without the need of expensive robotics or conveyor belts as used by more advanced solutions such as Phenopsis [38]. rosettR can be used to perform a primary screen of growth phenotypes that nevertheless require further validation in less artificial conditions such as soil experiments or field trials.

While being space efficient for performing the experiment, the plates have obvious growing constraints: in an experiment with long duration the roots are not allowed to develop freely and the leaves will become too big and overlap with each other. For that reason, rosettR experiments are limited to young seedlings; from our experience, after approximately 20 days the leaves started overlapping, but this obviously depends on the specific genotypes to be tested. For more flexible, yet less automated, analysis of rosette area where data sharing is not a concern, the online tool Phenophyte may serve as a good alternative platform [39].

Another important limitation of our protocol comes from the use of a 2D top-view of the seedlings which implies that traits such as plant height, leaf thickness, curvature, leaf angle or other morphology cannot be taken into account. Using rosettR, curved leaves will therefore result in smaller detected rosette area even when overall growth is unaffected. It is therefore important to carefully inspect seedlings for such characteristics, and where relevant use more high resolution approaches such as Phytotyping4D [24] or image analysis tools for flattened detached leaves [19, 21–23]. Phenotypes such as chlorosis or reduced chlorophyll concentration may be studied using other specialized tools [40]. As the seedlings are visible against a sterile white background, the thresholding for plant detection from the images is straight-forward yet not easily adapted to more complex backgrounds such as soil grown plants. For this, other non-destructive methods such as Easy Leaf Area [22] might be more appropriate.

The number of treatments that can be combined with the rosettR protocol is relatively high, particularly when compared to soil based systems where the application of certain chemicals at a large scale is difficult. The duration and intensity of any applied treatment is very much dependent on the goal of each individual experiment and needs to be optimized prior to performing large screens. For example, when applying a stress treatment by medium supplementation it is important to consider whether that supplement is stable for the whole duration of the experiment. If that is not the case, one might need to re-apply the treatment by transferring the seedlings to new plates using a membrane (see “Methods”). The use of a pilot experiment is highly recommended in order to obtain a good estimate of the expected variation between genotypes and/or treatments, and will provide a good indication on the number of biological replicates to use depending on the expected effect.

With the increasing adoption of high-level programming languages such as R and python by the community of plant scientists, we believe that bundling experimental protocols with the software needed to analyze and present the data is valuable as it facilitates performing the experiments, and at the same time avoiding the common data analysis bottleneck.

## Conclusions

 In this paper we presented rosettR, an experimental protocol and accompanying software for the measurement and analysis of total rosette areas of seedlings grown in tissue culture plates. rosettR is unique in its combination of being a high-throughput but still inexpensive and easy to use phenotyping platform. We demonstrated that treatment effects such as the response to different light regimes and osmotic stress, as well as differences between genotypes, could be readily detected. The rosettR R-package provides an efficient and affordable screening platform for growth related phenotypes with only minimal requirements on programming skills and equipment. rosettR is an open resource and the community is encouraged to contribute.
